# Changing epidemiological patterns of HIV and AIDS in China in the post-SARS era identified by the nationwide surveillance system

**DOI:** 10.1186/s12879-018-3551-5

**Published:** 2018-12-27

**Authors:** Zhenqiu Liu, Oumin Shi, Qiong Yan, Qiwen Fang, Jialu Zuo, Yue Chen, Xingdong Chen, Tiejun Zhang

**Affiliations:** 1 State Key Laboratory of Genetic Engineering and Collaborative Innovation Center for Genetics and Development, School of Life Sciences, Fudan University, Shanghai, 200032 China; 2 Department of Epidemiology, School of Public Health, Fudan University, Shanghai, China; 3 Key Laboratory of Public Health Safety (Fudan University), Ministry of Education, Shanghai, China; 4 Fudan University Taizhou Institute of Health Sciences, Taizhou, China; 5 Shenzhen Second People’s Hospital, the First Affiliated Hospital of Shenzhen University, Shenzhen, China; 6 Department of Child and Maternal Health, School of Public Health, Fudan University, Shanghai, China; 7 School of Epidemiology and Public Health Faculty of Medicine, University of Ottawa, Ottawa, Canada; 8 Human Phenome Institute, Fudan University, 825 Zhangheng Road, Shanghai, 201203 China

**Keywords:** HIV and AIDS, China, Surveillance, Incidence, Epidemiology

## Abstract

**Background:**

China has made substantial progress in tackling its HIV and AIDS epidemic. But the changing patterns of HIV and AIDS incidence based on the longitudinal observation data were rarely studied.

**Methods:**

The reporting incidence (RI) and mortality data on HIV and AIDS in China covering 31 provinces from 2004 to 2014 were collected from the Chinese Public Health Science Data Center. To decompose the time-series data, Empirical Mode Decomposition (EMD) was applied to properly describe the trends of HIV and AIDS incidence. A mathematical model was used to estimate the relative change of incidence among provinces and age groups.

**Results:**

A total of 483,010 newly HIV infections and 214,205 AIDS cases were reported between 2004 and 2014 nationwide. HIV infection increased from 13,258 in 2004 (RI 1.02 per 100,000 person years) to 74,048 in 2014 (RI 5.46 per 100,000). The number of AIDS cases increased from 3054 in 2004 (RI 0.23 per 100,000) to 45,145 in 2014 (RI 3.33 per 100,000). The overall relative changes for HIV infection and AIDS incidence were 1.11 (95% confidence interval [CI] 1.10–1.13) and 1.28 (95% CI 1.23–1.33), respectively. The relative increase for HIV and AIDS RI was higher in northwest provinces while lower in Henan, Xinjiang, Guangxi and Yunnan. The overall relative changes for HIV infection were 1.12 (95% CI 1.11–1.14) in males and 1.10 (95% CI 1.06–1.13) in females. For AIDS RI, the relative increases were 1.31 (95% CI 1.26–1.36) in males and 1.22 (95% CI 1.17–1.28) in females. The lowest relative increase was detected among young adults, while the largest relative increase (odds ratio [OR] > 1.30) was detected in people aged 55 years or above.

**Conclusions:**

HIV and AIDS showed an increasing trend in China from 2004 to 2014, respectively, but the epidemic tended to be under control among provinces and young people that used to have a high HIV and AIDS incidence. Northwest China and older people could be new “hop-spots” for HIV and AIDS risk.

## Background

Although great efforts have been made by Chinese government over the last three decades, HIV and AIDS remains an important public health concern in China [1, 2]. In 2014, more than half a million people living with HIV and 0.2 million AIDS cases were reported, and there were more than 0.1 million new HIV infections [3]. Since the first AIDS case was reported in early 1980’s, China had initiated a set of measures to combat this emerging health problem. HIV and AIDS surveillance started in 1985. In 1995, the China Ministry of Health and the National Center for AIDS established 42 national sentinel sites in 23 of the 31 provinces [4]. However, the system had issues related to its accessibility and accuracy, which hampered timely understanding of the HIV and AIDS epidemic pattern and effectiveness of HIV and AIDS prevention measures.

The outbreak of severe acute respiratory syndromes (SARS) in 2003 revealed some shortcomings of China’s infectious disease prevention system and triggered a rapid mobilization of public health policies for a broader range of health challenges. In particular, the HIV response has been strengthened by an influx of new funds and political support such as the “Four Free and One Care” policy [5]. A web-based HIV reporting system was established in 2005 and integrated the HIV and AIDS surveillance system [6], which provides a unique opportunity to comprehensively understand the epidemiological features of HIV and AIDS in China.

According to a recent study based on data collected from a routine infectious disease reporting system, ~ 20% (10/45) infectious diseases showed a significantly increasing trend in incidence from 2004 to 2013. HIV infection showed the fastest growth with annual percentage change of 16.3% in reporting incidence [7], but the study did not provide province-specific and age-specific results.

To better understand the epidemiological characteristics of HIV and AIDS in China, we used the surveillance data, by province, sex, and age, to determine the temporal trend of HIV and AIDS incidence in the country from 2004 to 2014, and the changing patterns of HIV and AIDS incidence across provinces and age groups. Moreover, we collected the mortality data to assess the HIV and AIDS disease burden in the same period. All these will assist evidence-based and region-specific planning and evaluation of the effectiveness of current treatment and prevention strategies, as well as the assessment of future tendency and subsequent allocation of limited health resources.

## Methods

### Data collection

A routine reporting system for selected infectious diseases covered 31 provinces in mainland China was established by the Chinese government in 1950s [8]. It has become a web-based system since 2003. The number of notifiable infectious diseases in this system increased from 18 before 1978 to 39 after 2003. The notifiable infectious diseases were divided into Classes A, B and C. According to the Infectious Disease Prevention Act, all epidemic reports are time-sensitive. All Class A infectious diseases as well as pulmonary anthrax and SARS in Class B should be reported through the system within 2 h after diagnosis, and other disease in Class B and Class C infectious diseases should be reported within 24 h after diagnosis. As a result, the date of diagnosis and reporting is the same day. We extracted HIV and AIDS data from 2004 and 2014 from the Science Data Center of Public Health (http://www.phsciencedata.cn). AIDS patients in our study were those who had a previous HIV diagnosis, which had been identified or not, and then progressed to late stage.

### Statistical analysis

#### Incident trend decomposition

Empirical mode decomposition (EMD), an adaptive model for non-liner and non-stationary time series data [9], was used to identify the temporal trend of HIV and AIDS incidence from 2004 to 2014. The EMD method decomposed original data into several oscillatory components, corresponding to some physical phenomenon underlying the data, and the residue of decomposition is the unbiased surrogate of the true trend of the data [10]. The physical phenomenon varied across different scenarios and therefore hardly to be specified here. For example, if the data were time series of HIV incidence, the physical phenomenon included the effect size of infected people, the using rate of condom, and the HIV/AIDS polices etc.

#### Patterns of time changes in incidence and mortality of HIV and AIDS

In order to investigate the changing patterns of HIV and AIDS epidemic and to specify a mathematical model that allows comparisons among sexes, different provinces and age groups [11], we processed the original incidence and mortality data with a logit transformation and then assessed secular changes via polynomial and spline models (in our surveillance system, the incidence and mortality rate was calculated by population as denominator). As linear trends were observed in most provinces and age groups, a linear model was applied for incidence data on the logit scale. Relative changes in HIV and AIDS incidence or mortality were presented by odds ratio (OR) as compared with 2004. In addition, we also calculated average annual percentage change (AAPC) in HIV and AIDS RIs [12]. All analyses were conducted using R (version 3.3.3).

In the current analysis, “HIV infection” referred to the presence of HIV infection at the time of reporting. “AIDS” cases were newly diagnosed AIDS patients.

## Results

### Temporal trends of HIV and AIDS incidence

**Fig. 1:**
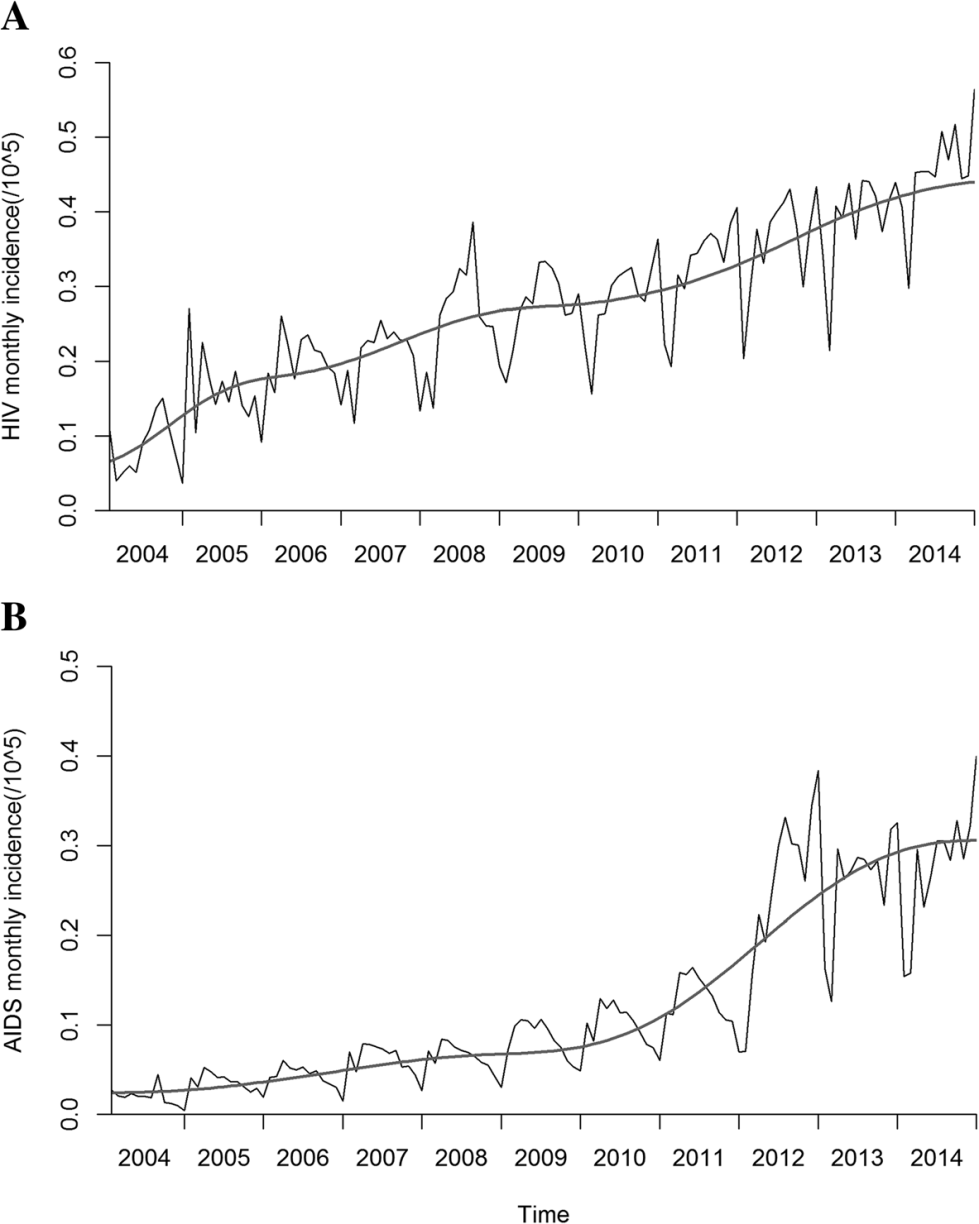
Monthly incidence of HIV and AIDS in China from 2004 to 2014. The gray smooth lines plotted in Fig. 1 were obtained from residues of EMD. (**a**: HIV monthly incidence from Jan, 2004 to Dec, 2014; **b**: AIDS monthly incidence from Jan, 2004 to Dec, 2014)

During the period of 2004–2014, a total of 483,010 newly HIV infections and 214,205 AIDS cases were reported nationwide. Reported HIV infection cases increased from 13,258 in 2004 (RI: 1.02 per 100,000) to 74,048 in 2014 (RI = 5.46 per 100,000). Reported AIDS cases increased from 3054 in 2004 (RI = 0.23 per 100,000) to 45,145 in 2014 (RI = 3.33 per 100,000). The overall relative changes in incidence measured by OR were 1.11 (95% CI 1.10–1.13) for HIV and 1.28 (95% CI 1.23–1.33) for AIDS, respectively. The increase for HIV infection incidence was in a linear fashion. For AIDS incidence, the linear increase was smaller for the period of 2004–2009, with a relative change of 1.06 (95% CI 1.03–1.09), it was larger between 2010 and 2013, with a relative change of 1.44 (95% CI 1.37–1.50). The incidence of reported AIDS was plateaued in 2014 (Fig. 1).

### Changing patterns of HIV and AIDS incidence according to each province

**Fig. 2:**
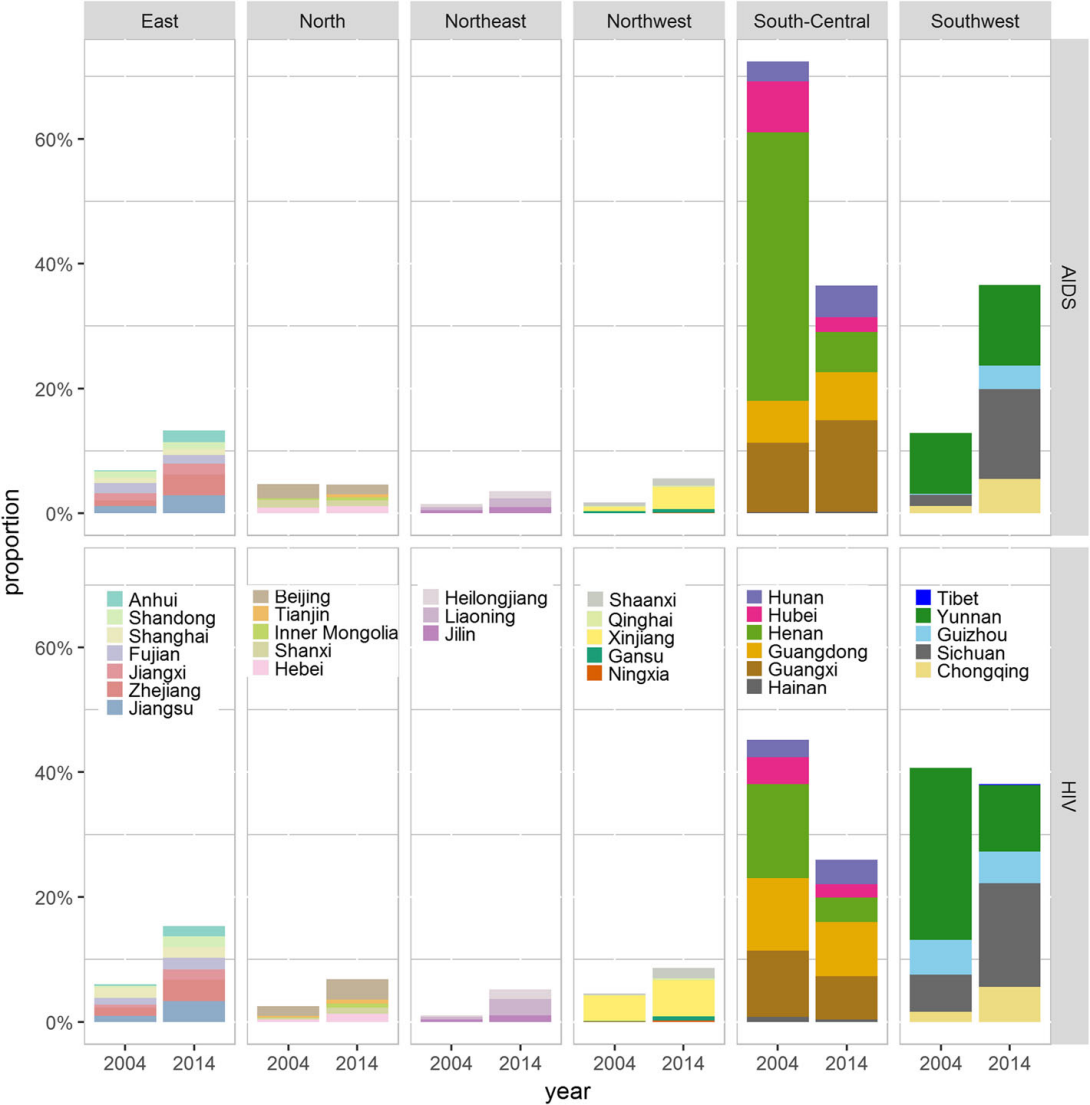
The distribution of HIV and AIDS cases by province in 2004 and 2014

**Fig. 3:**
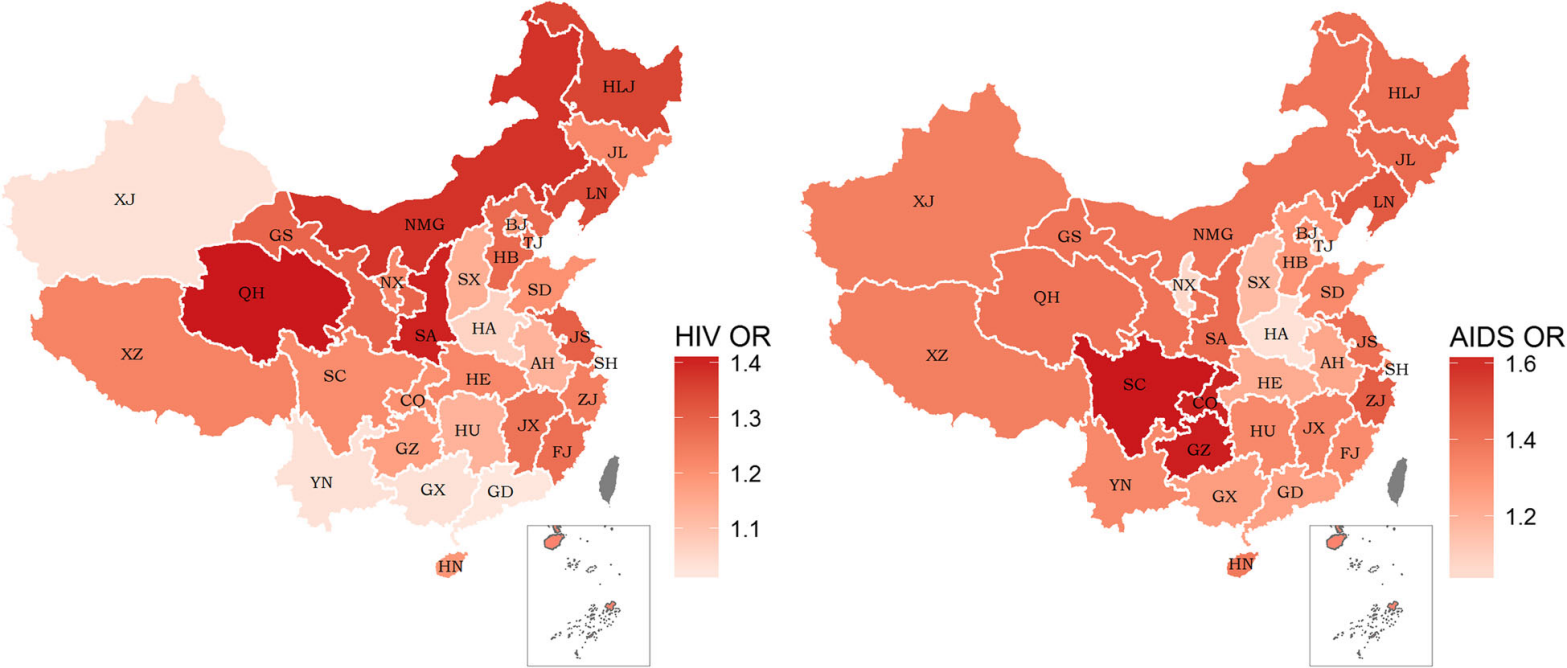
The relative change (odds ratio) of HIV and AIDS incidences in China from 2004 to 2014. (The maps were derived from *ggmap* package in R program. BJ: Beijing, TJ: Tianjin, HB: Hebei, NMG: Inner Mongolia, SX: Shanxi, HLJ: Heilongjiang, JL: Jilin, LN: Liaoning, SD: Shandong, JS: Jiangsu, SH: Shanghai, ZJ: Zhejiang, AH: Anhui, FJ: Fujian, JX: Jiangxi, GD: Guangdong, GX: Guangxi, HN: Hainan, YN: Yunnan, GZ: Guizhou, XZ: Tibet, SC: Sichuan, CQ: Chongqing, HU: Hunan, HE: Hubei, HA: Henan, SA: Shaanxi, GS: Gansu, NX: Ningxia, QH: Qinghai, XJ: Xinjiang)

The distributions of HIV infection and AIDS varied geographically in China (Fig. 2; Figs. 1 and 2 in Appendix). An increasing trend for both HIV infection and AIDS RIs was observed in all provinces from 2004 to 2014 (OR > 1.00) (Table 1; Fig. 3). In 2004, four provinces including Yunnan, Guangxi, Xinjiang and Henan, had the highest HIV incidence, and accounted for 56.1% of all newly HIV infections (Fig. 2), with the ORs being 1.13 (95% CI 1.09–1.16), 1.06 (95% CI 1.03–1.08), 1.03 (95% CI 1.01–1.05) and 1.06 (95% CI 1.02–1.10), respectively. In 2014, four provinces with the highest HIV incidence were Xinjiang, Yunnan, Sichuan (OR = 1.21, 95% CI 1.15–1.28) and Chongqing (OR = 1.20, 95% CI 1.16–1.25), and the incidence was all above 14.0 per 100,000. The highest relative increase in the incidence of HIV infection occurred in Shaanxi (OR = 1.40, 95% CI 1.33–1.47), Qinghai (OR = 1.41, 95% CI 1.33–1.49) and Inner Mongolia (OR = 1.38, 95% CI 1.33–1.44), which were all located in North and Northwest China.

**Table 1:** The relative change of HIV and AIDS incidence in each province in China from 2004 to 2014

Region	Province	HIV incidence in 2004 (/10^5)	Relative Changes of HIV incidence			AIDS incidence in 2004 (/10^5)	Relative Changes of AIDS incidence		
			OR	95%CI			OR	95%CI	
	ALL	1.02	1.11	1.10	1.13	0.23	1.28	1.23	1.33
North	Beijing	1.38	1.18	1.14	1.22	0.48	1.32	1.20	1.45
	Tianjin	0.37	1.26	1.18	1.33	0.00	1.09	1.07	1.12
	Hebei	0.08	1.28	1.24	1.31	0.04	1.29	1.20	1.37
	Shanxi	0.12	1.14	1.09	1.18	0.11	1.16	1.09	1.23
	Inner Mongolia	0.05	1.38	1.33	1.44	0.03	1.40	1.25	1.56
Northeast	Liaoning	0.11	1.34	1.29	1.40	0.04	1.48	1.40	1.58
	Jilin	0.23	1.22	1.17	1.28	0.06	1.43	1.38	1.49
	Heilongjiang	0.11	1.35	1.30	1.41	0.04	1.42	1.37	1.47
East	Shanghai	1.62	1.06	1.02	1.10	0.19	1.34	1.24	1.46
	Jiangsu	0.17	1.30	1.25	1.35	0.05	1.41	1.33	1.48
	Zhejiang	0.39	1.24	1.19	1.28	0.06	1.47	1.42	1.53
	Anhui	0.05	1.13	1.05	1.21	0.01	1.23	1.13	1.33
	Fujian	0.40	1.27	1.23	1.32	0.14	1.32	1.28	1.36
	Jiangxi	0.14	1.26	1.20	1.32	0.08	1.35	1.30	1.40
	Shandong	0.03	1.20	1.14	1.27	0.03	1.32	1.20	1.45
Central	Henan	2.06	1.06	1.02	1.10	1.36	1.03	1.01	1.05
	Hubei	0.93	1.22	1.19	1.24	0.41	1.21	1.14	1.28
	Hunan	0.57	1.13	1.10	1.15	0.14	1.33	1.28	1.38
South	Guangdong	1.94	1.04	1.01	1.06	0.26	1.25	1.20	1.31
	Guangxi	2.90	1.06	1.03	1.08	0.70	1.26	1.19	1.34
	Hainan	1.31	1.19	1.16	1.22	0.05	1.38	1.23	1.55
Southwest	Chongqing	0.71	1.20	1.16	1.25	0.11	1.62	1.54	1.70
	Sichuan	0.89	1.21	1.15	1.28	0.06	1.64	1.54	1.75
	Guizhou	1.93	1.17	1.13	1.21	0.01	1.63	1.46	1.82
	Yunnan	8.39	1.13	1.09	1.16	0.68	1.33	1.23	1.44
	Tibet	0.00	1.23	1.16	1.35	0.00	1.36	1.26	1.47
Northwest	Shaanxi	0.09	1.40	1.33	1.47	0.05	1.43	1.37	1.50
	Gansu	0.07	1.29	1.23	1.34	0.04	1.40	1.21	1.61
	Qinghai	0.17	1.41	1.33	1.49	0.13	1.40	1.27	1.54
	Ningxia	0.14	1.22	1.18	1.26	0.00	1.06	1.04	1.08
	Xinjiang	2.84	1.03	1.01	1.05	0.10	1.36	1.24	1.50

In 2004, Henan, Guangxi, Yunnan and Beijing had the top 4 AIDS incidence rates (Fig. 1 in Appendix). Henan was the only province where AIDS incidence exceeded 1.0 per 100,000 from 2004 to 2014, but had the lowest relative increase (OR = 1.03, 95%CI 1.01–1.05). In 2014, Guangxi, Yunnan, Chongqing and Sichuan, all in Southwest China, had the highest AIDS incidence rates (Fig. 2; Fig. 1 in Appendix). Sichuan had the largest relative increase (OR = 1.64, 95% CI 1.54–1.75), followed by Guizhou (OR = 1.63, 95% CI 1.46–1.82) and Chongqing (OR = 1.62, 95% CI 1.54–1.70), which were all in Southwest China (Table 1; Fig. 3).

### Changing patterns of HIV and AIDS incidence stratified by age

**Fig. 4:**
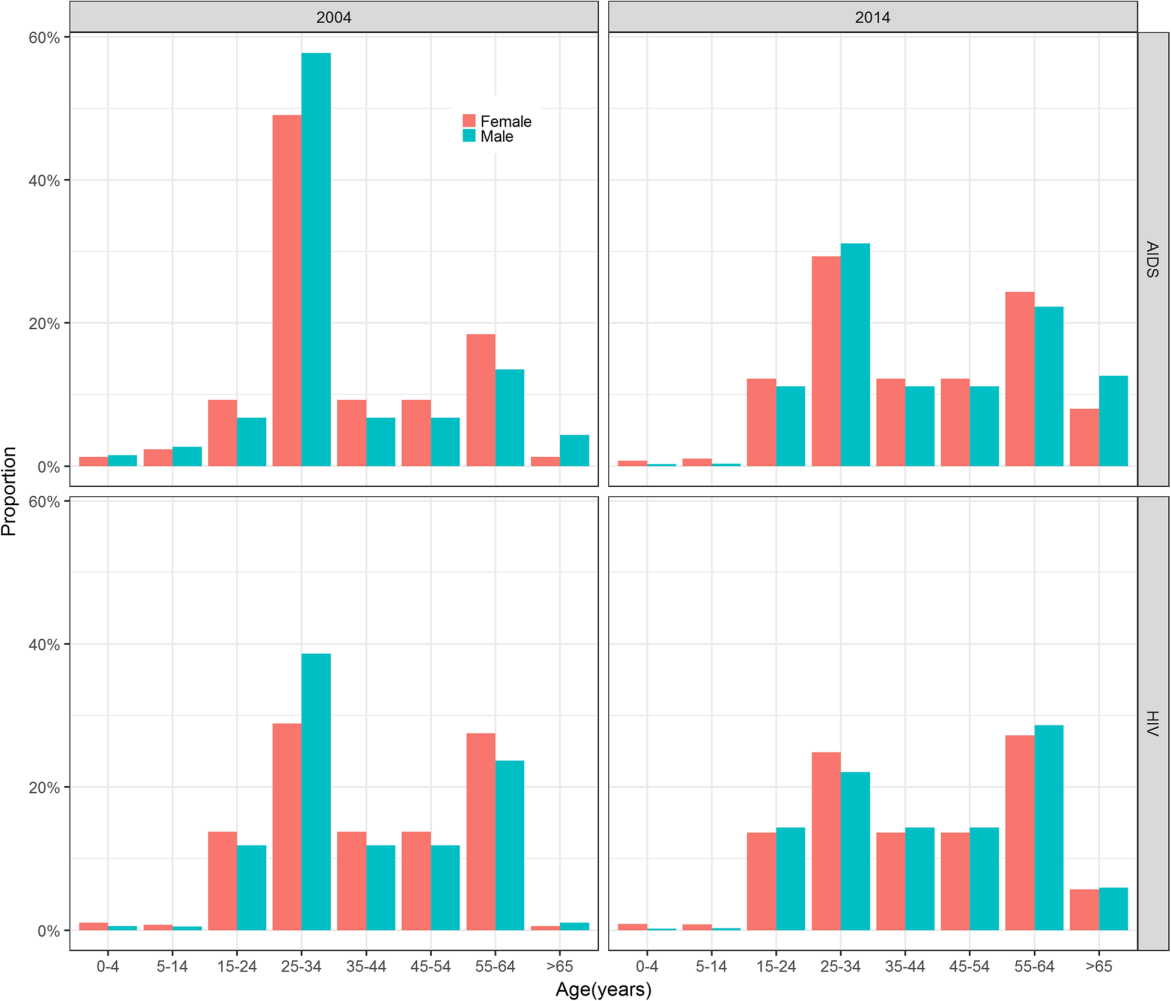
The distribution of HIV and AIDS cases by age in 2004 and 2014

The age-specific incidences of HIV infection and AIDS changed over time. In 2004, adults aged 25–34 years accounted for 36.04% of all new HIV infections and 54.78% of all new diagnosed AIDS cases. In 2014, the corresponding proportions were 22.58 and 30.69%, respectively. However, the numbers of newly infected HIV and diagnosed AIDS cases unexpectedly increased among people aged 55 years or above between 2004 and 2014 (Fig. 4). In order to further investigate the HIV and AIDS changing patterns associated with age, we divided age into 18 groups stratified by gender.

**Fig. 5:**
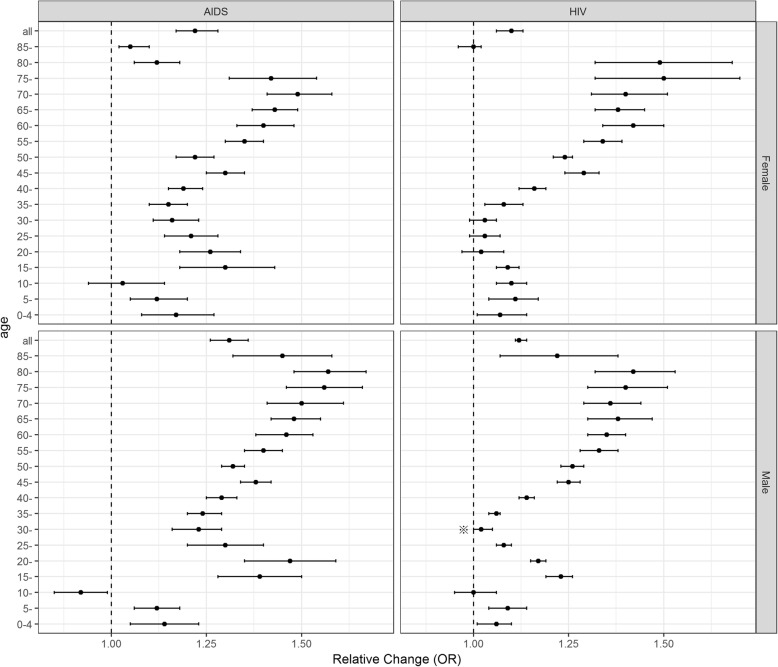
The relative change (odds ratio) of HIV and AIDS incidence in each age group stratified by gender from 2004 to 2014. (※: *p* < 0.05)

The overall relative changes of reported HIV incidence among males and females were 1.12 (95% CI 1.11–1.14) and 1.10 (95% CI 1.06–1.13), respectively. Males and females had a similar changing pattern of HIV incidence (Table 2). Most age groups experienced an increase in HIV infection from 2004 to 2014 (Fig. 5).

**Table 2:** The relative change of HIV incidence in different age group in China from 2004 to 2014

Age group	Relative Change				Male	Relative Change			Female	Relative Change		
	HIV incidence in 2004 (/10^5)	OR	95%CI		HIV incidence in 2004 (/10^5)	OR	95%CI		HIV incidence in 2004 (/10^5)	OR	95%CI	
All	0.99	1.12	1.10	1.13	1.46	1.12	1.11	1.14	0.51	1.10	1.06	1.13
0-	0.15	1.07	1.01	1.12	0.16	1.06	1.01	1.10	0.14	1.07	1.01	1.14
5-	0.08	1.10	1.05	1.15	0.09	1.09	1.04	1.14	0.06	1.11	1.04	1.17
10-	0.06	1.04	0.99	1.08	0.07	1.00	0.95	1.06	0.04	1.10	1.06	1.14
15-	0.38	1.18	1.16	1.20	0.45	1.23	1.19	1.26	0.29	1.09	1.06	1.12
20-	1.33	1.12	1.10	1.14	1.88	1.17	1.15	1.19	0.76	1.02	0.97	1.08
25-	2.86	1.07	1.05	1.08	4.35	1.08	1.06	1.10	1.36	1.03	0.99	1.07
30-	3.21	1.02	1.01	1.04	5.02	1.02	1.00	1.05	1.33	1.03	0.99	1.06
35-	1.84	1.06	1.04	1.08	2.81	1.06	1.04	1.07	0.81	1.08	1.03	1.13
40-	1.02	1.14	1.12	1.17	1.49	1.14	1.12	1.16	0.52	1.16	1.12	1.19
45-	0.62	1.26	1.22	1.29	0.91	1.25	1.22	1.28	0.32	1.29	1.24	1.33
50-	0.55	1.25	1.22	1.28	0.67	1.26	1.23	1.29	0.41	1.24	1.21	1.26
55-	0.23	1.33	1.28	1.38	0.28	1.33	1.28	1.38	0.19	1.34	1.29	1.39
60-	0.17	1.37	1.31	1.43	0.24	1.35	1.30	1.40	0.10	1.42	1.34	1.50
65-	0.18	1.38	1.31	1.46	0.28	1.38	1.30	1.47	0.06	1.38	1.32	1.45
70-	0.12	1.37	1.29	1.45	0.20	1.36	1.29	1.44	0.04	1.40	1.31	1.51
75-	0.07	1.41	1.30	1.52	0.13	1.40	1.30	1.51	0.02	1.50	1.32	1.70
80-	0.04	1.42	1.32	1.54	0.07	1.42	1.32	1.53	0.01	1.49	1.32	1.68
85-	0.25	1.17	1.01	1.36	0.56	1.22	1.07	1.38	0.06	1.00	0.96	1.02

In males, the highest incidence of HIV infection in 2004 was found in people aged 30–34 years (5.02 per 100,000), but the relative increase was low (OR = 1.02, 95% CI 1.00–1.05, *p* = 0.027). The largest relative increase was observed in men aged 80–84 (OR = 1.42, 95% CI 1.32–1.53), albeit the lowest incidence in 2004 (0.07 per 100,000). The highest incidence of HIV infection in 2004 was found in women aged 25–29 years (1.36 per 100,000), but the incidence remained stable in the study period (OR = 1.03, 95% CI 0.99–1.07). The highest relative increase was detected in women aged 75–79 (OR = 1.50, 95% CI 1.32–1.70) with the HIV infection incidence being 0.02 per 100,000 in 2004.

For AIDS incidence, the relative increases in males and females were 1.31 (95% CI 1.26–1.36) and 1.22 (95% CI 1.17–1.28), respectively (Table 3; Fig. 5). All age groups except the age group of 10–14 years showed an increasing trend. In 2004, the highest AIDS incidence was found in people aged 30–34 years in both genders, but the relative increase was low for both males (OR = 1.23, 95% CI 1.16–1.29) and females (OR = 1.16, 95% CI 1.11–1.23). The largest relative increase was observed in older people (age ≥ 55 years) for both sexes. In males, the largest OR was observed in the age group of 80–84 years (OR = 1.57, 95% CI 1.48–1.67). In females, the largest OR was detected in the age group of 70–74 years (OR = 1.49, 95% CI 1.41–1.58).

**Table 3:** The relative change of AIDS incidence in different age group in China from 2004 to 2014

Age group	Both sex	Relative Change			Male	Relative Change			Female	Relative Change		
	AIDS incidence in 2004 (/10^5)	OR	95%CI		AIDS incidence in 2004 (/10^5)	OR	95%CI		AIDS incidence in 2004 (/10^5)	OR	95%CI	
All	0.23	1.28	1.23	1.34	0.29	1.31	1.26	1.36	0.16	1.22	1.17	1.28
0-	0.04	1.15	1.06	1.25	0.05	1.14	1.05	1.23	0.02	1.17	1.08	1.27
5-	0.04	1.12	1.07	1.18	0.05	1.12	1.06	1.18	0.03	1.12	1.05	1.20
10-	0.03	0.96	0.89	1.04	0.03	0.92	0.85	0.99	0.02	1.03	0.94	1.14
15-	0.02	1.35	1.24	1.47	0.02	1.39	1.28	1.50	0.01	1.30	1.18	1.43
20-	0.10	1.38	1.29	1.47	0.11	1.47	1.35	1.59	0.08	1.26	1.18	1.34
25-	0.39	1.27	1.19	1.35	0.54	1.30	1.20	1.40	0.24	1.21	1.14	1.28
30-	0.65	1.21	1.15	1.27	0.88	1.23	1.16	1.29	0.41	1.16	1.11	1.23
35-	0.51	1.22	1.17	1.27	0.64	1.24	1.20	1.29	0.38	1.15	1.10	1.20
40-	0.40	1.26	1.22	1.30	0.48	1.29	1.25	1.33	0.32	1.19	1.15	1.24
45-	0.25	1.36	1.32	1.40	0.31	1.38	1.34	1.42	0.19	1.30	1.25	1.35
50-	0.27	1.29	1.25	1.32	0.33	1.32	1.29	1.35	0.20	1.22	1.17	1.27
55-	0.13	1.38	1.34	1.43	0.17	1.40	1.35	1.45	0.09	1.35	1.30	1.40
60-	0.12	1.44	1.37	1.51	0.16	1.46	1.38	1.53	0.06	1.40	1.33	1.48
65-	0.08	1.47	1.41	1.53	0.13	1.48	1.42	1.55	0.02	1.43	1.37	1.49
70-	0.07	1.50	1.41	1.60	0.12	1.50	1.41	1.61	0.01	1.49	1.41	1.58
75-	0.03	1.54	1.44	1.64	0.05	1.56	1.46	1.66	0.01	1.42	1.31	1.54
80-	0.01	1.58	1.49	1.68	0.02	1.57	1.48	1.67	0.00	1.12	1.06	1.18
85-	0.01	1.45	1.31	1.60	0.04	1.45	1.32	1.58	0.00	1.05	1.02	1.10

### Changing patterns of HIV and AIDS mortality

Overall, the mortality rate for HIV and AIDS in China was increasing (Fig. 3 and Table 1 in Appendix). The relative increase was 1.29 (95% CI 1.18–1.40) nationwide. Geographically, the largest OR was found in Xinjiang (OR = 1.64, 95% CI 1.37–1.97). Henan was the only province where HIV and AIDS mortality remained stable from 2004 to 2014 (OR = 1.07, 95% CI 0.96–1.19). Moreover, HIV and AIDS mortality showed an increasing trend among all age groups except for the age group of 20–24 years in both genders (Table 2 in Appendix).

The results of AAPC were presented in Tables 3, 4, 5 and 6 in Appendix. No significant difference was detected.

## Discussion

Since the first report of HIV and AIDS case in China, the HIV and AIDS epidemic has been changing both geographically and temporally [13–19]. In this study, we provided a comprehensive overview of HIV and AIDS epidemic in China, and assessed secular changes among provinces and age groups for the period of 2004–2014. The lowest relative increase of HIV and AIDS incidence was detected in provinces with a high HIV and AIDS incidence in 2004. Some Northwestern provinces such as Qinghai and Shaanxi had the strongest relative increase of HIV and AIDS incidence. In addition, young men and women aged 20–34 years who had the highest HIV and AIDS incidence in 2004 experienced the lowest relative increase during the study period. Older men and women (age ≥ 55 years) showed the largest increase of HIV and AIDS incidence.

The HIV and AIDS surveillance/reporting system has been improving, which may explain part of the observed increases of HIV and AIDS incidence and mortality. China has more than 1800 sentinel sites for HIV and AIDS surveillance [15]. The counseling and testing services have been continuously strengthened. By the end of 2014, there were 455 HIV testing and confirmation labs and 25,762 screening labs in China, covering 96.5% of the country [20], and 21,210 medical and health institutions conducted ~ 130 million person-times of HIV antibody tests, increasing from 84 million in 2011 [20].

China has achieved a substantial progress in combatting with HIV and AIDS. For instance, AIDS incidence showed a plateau after 2013, which might imply a near-future decrease of AIDS cases with the persistent expansion of availability of highly active anti-retroviral therapy (HAART) [21]. Also, the control of HIV and AIDS epidemic in Henan, Xinjiang, Guangxi and Yunnan might be an indication for the effectiveness of preventive strategies and measures after the SARS outbreak, and these areas used to be the “hot-spots” of HIV and AIDS in China [22].

In the first two decades of HIV and AIDS era in China, Henan, Guangxi, Yunnan and Xinjiang had high HIV and AIDS prevalence [23], but had different transmission modes. After dramatically cracking down the illegal blood trade in Henan [24, 25], HIV and AIDS incidence showed the lowest relative change there. Guangxi and Yunnan are close to the “Golden Triangle” in Burma, where intravenous drug use was once the major source of HIV transmission, but unprotected sex was found to be a main source of HIV transmission recently [26, 27]. In this study, Guangxi and Yunnan had a relatively slow increasing trend for both HIV infection and AIDS incidence, which might ascribe to substantial targeted endeavors combating HIV and AIDS in the last decade. A similar changing pattern was observed in Xinjiang, where the HIV and AIDS incidence was high in 2004, but the relative increase was low during the study period.

However, it should be noted that some new HIV and AIDS “hot-spots” including Sichuan and Chongqing have appeared recently according to our analyses. Additionally, Inner Mongolia, Qinghai and Shaanxi showed great relative increases in the HIV and AIDS incidence. We should pay much more attention to the prevention and control of HIV and AIDS in these provinces.

There were also substantial changes in HIV and AIDS incidence associated with age. Men and women aged 20–34 years had the lowest relative increase, while those aged 55 years or above had the highest relative increase. The results suggested the effectiveness of measures that focused on young adults but also indicated a potential upsurge of HIV and AIDS epidemic among older people. Globally, previous evidence has suggested a considerable burden of HIV and AIDS among older adults [28–30]. Thus, it is important to further investigate the risk factors of HIV infection and to prioritize the prevention and control of HIV and AIDS among older adults.

Surprisingly, an increased mortality of HIV and AIDS was detected in the present study. We speculate that the increase of HIV and AIDS mortality may be partially explained by the accumulation of AIDS cases, since the cumulative AIDS-related mortality rates significantly increased over time after diagnosis [31]. Meanwhile, the inadequate provision of treatment services and difficulty with adherence were also likely to contribute to the increased mortality, particularly before 2003. Therefore, the accumulation in HIV infections and improved survival time among AIDS patients could result in an increasing burden of HIV and AIDS [32, 33].

Some limitations of our study should be mentioned. First, since the data were retrieved from a passive surveillance system, there might be some under reporting, especially during earlier years of the system. Second, the incidence could also be underestimated because of ascertainment bias by self-selection that individuals at high risk of HIV and AIDS were more reluctant to screening. Third, the increase of HIV and AIDS reporting incidence among older adults might be ascribed to the detection of long-standing infections, though more investigations should be warranted in the near future. Finally, key affected populations (e.g. the men who have sex with men) have not been particularized in our study due to the inaccessibility of corresponding surveillance data.

Estimation of the incidence of most infectious diseases is challenging, because infection might have occurred several years before symptoms arise or a diagnosis is made [34, 35]. Fortunately, the surveillance data can serve as good proxy to quantify the temporal trends of infectious diseases. The China Information System for Disease Control and Prevention (CISDCP) is currently the largest web-based surveillance system in the world, and it stores the nationwide validated reporting data daily. Despite these challenges, our analysis is based on a large sample of people diagnosed with HIV and AIDS across 31 provinces and over 11 years, which strengthens the reliability of our findings.

## Conclusions

We used a longitudinal surveillance dataset spanning 11 years in China to investigate changes in the epidemiological characteristics of HIV and AIDS after SARS outbreak. HIV and AIDS incidence showed a significant increasing trend in the last decade, but the epidemic has been well controlled among provinces where the HIV and AIDS incidence were high, and among young adults. However, the major findings also highlight the unmet need for HIV and AIDS prevention efforts and call for a beforehand measure to prevent the emergency among certain provinces and elderly people.

## Figures and Tables

**Fig. 6:**
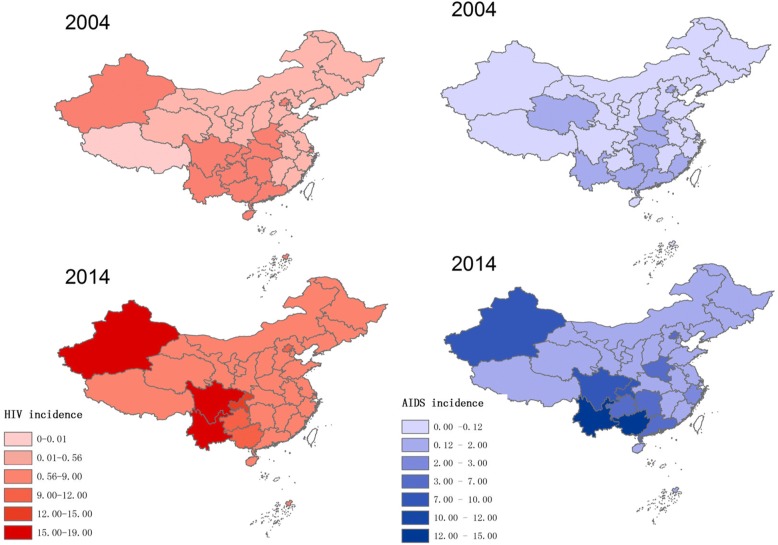
The HIV and AIDS incidence in China in 2004 and 2014

**Fig. 7:**
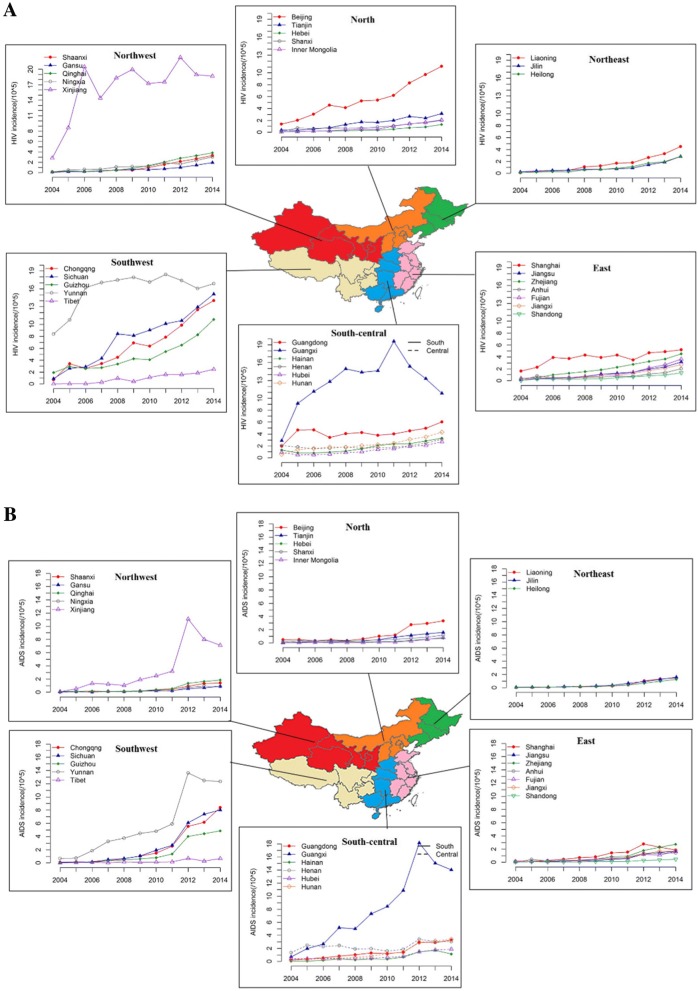
The incidence of HIV and AIDS in each province stratified by regions in China from 2004 to 2014. (**a**: HIV incidence in China at province level; **b**: AIDS incidence in China at province level)

**Fig. 8:**
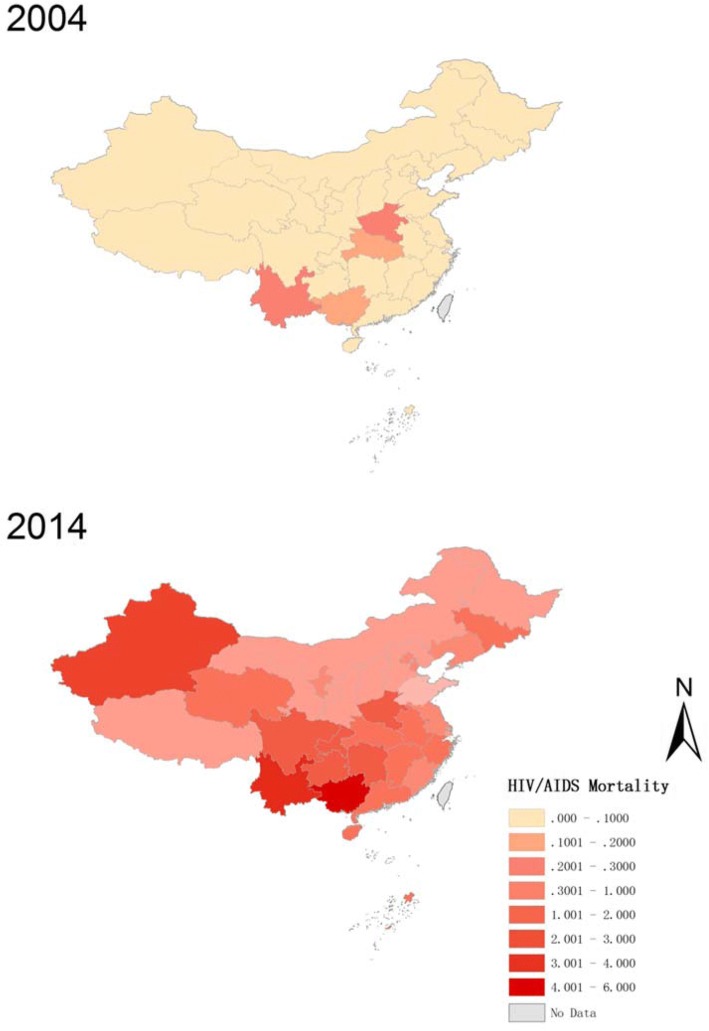
The HIV and AIDS mortality in China in 2004 and 2014

**Fig. 9:**
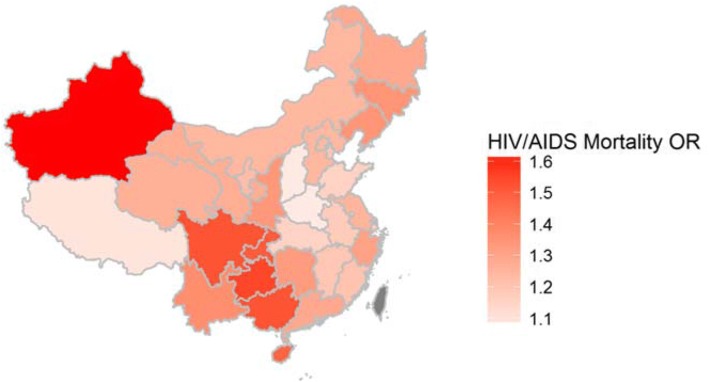
The relative change of mortality rate of HIV and AIDS in China at provincial level from 2004 to 2014

## Appendix

**Table 4:** The relative change of mortality rate of HIV and AIDS in China at province level from 2004 to 2014

Province	Mortality in 2004	Mortality rate in 2004 (/10^5)	OR	95%CI		*P* value
All	741	0.0570	1.29	1.18	1.40	< 0.001
Beijing	71	0.0135	1.23	1.15	1.32	< 0.001
Tianjin	0	0	1.19	1.16	1.23	< 0.001
Hebei	27	0.0088	1.24	1.08	1.42	0.0067
Shanxi	37	0.0060	1.07	1.02	1.12	0.013
Inner Mongolia	8	0.0126	1.24	1.12	1.38	0.002
Liaoning	15	0.0192	1.35	1.26	1.44	< 0.001
Jilin	16	0.0338	1.35	1.28	1.42	< 0.001
Heilongjiang	14	0.0133	1.29	1.23	1.36	< 0.001
Shanghai	25	0.0299	1.17	1.09	1.25	0.001
Jiangsu	35	0.0304	1.25	1.21	1.29	< 0.001
Zhejiang	28	0.0213	1.31	1.23	1.40	< 0.001
Anhui	6	0.0031	1.17	1.09	1.25	< 0.001
Fujian	52	0.0584	1.20	1.10	1.31	0.002
Jiangxi	34	0.0560	1.19	1.14	1.24	< 0.001
Shandong	31	0.0153	1.15	1.11	1.20	< 0.001
Henan	1315	0.2022	1.07	0.96	1.19	0.206
Hubei	248	0.1598	1.15	1.09	1.21	< 0.001
Hunan	9	0.0689	1.33	1.24	1.44	< 0.001
Guangdong	205	0.0629	1.29	1.18	1.40	< 0.001
Guangxi	341	0.1091	1.53	1.35	1.73	< 0.001
Hainan	4	0.0123	1.49	1.24	1.79	0.0011
Chongqing	34	0.0383	1.51	1.35	1.67	< 0.001
Sichuan	56	0.0252	1.53	1.35	1.73	< 0.001
Guizhou	5	0.0078	1.56	1.27	1.90	0.001
Yunnan	298	0.2411	1.37	1.20	1.57	< 0.001
Tibet	0	0	1.09	1.06	1.15	< 0.001
Shaanxi	17	0.0219	1.34	1.20	1.50	< 0.001
Gansu	11	0.0383	1.27	1.15	1.39	< 0.001
Qinghai	7	0.0185	1.26	1.22	1.30	< 0.001
Ningxia	0	0	1.22	1.18	1.26	< 0.001
Xinjiang	18	0.0269	1.64	1.37	1.97	< 0.001

**Table 5:** The relative change of mortality rate of HIV and AIDS in each age group in China from 2004 to 2014

age group	Mortality in 2004	Mortality rate in 2004 (/10^5)	OR	95%CI		*P* value
Male						
1 to 4	3	0.0505	1.05	1.03	1.07	< 0.001
5 to 9	5	0.0080	1.10	1.08	1.12	< 0.001
10 to 14	4	0.0061	1.17	1.15	1.18	< 0.001
15 to 19	4	0.0076	1.19	1.16	1.22	< 0.001
20 to 24	21	0.0438	0.98	0.97	1.00	0.072
25 to 29	70	0.1162	1.02	1.00	1.04	0.033
30 to 34	106	0.1622	1.04	1.03	1.06	< 0.001
35 to 39	102	0.1817	1.08	1.06	1.10	< 0.001
40 to 44	82	0.1941	1.09	1.07	1.11	< 0.001
45 to 49	36	0.0819	1.08	1.05	1.11	< 0.001
50 to 54	31	0.0945	1.08	1.06	1.11	< 0.001
55 to 59	22	0.0914	1.07	1.04	1.10	< 0.001
60 to 64	14	0.0655	1.07	1.05	1.10	< 0.001
65 to 69	8	0.0456	1.07	1.05	1.10	< 0.001
70 to 74	5	0.0402	1.07	1.04	1.10	< 0.001
75 to 79	0	0	1.07	1.04	1.09	< 0.001
80 plus	0	0	1.08	1.05	1.11	< 0.001
Female						
1 to 4	3	0.0096	1.05	1.03	1.07	< 0.001
5 to 9	2	0.0048	1.10	1.09	1.12	< 0.001
10 to 14	4	0.0067	1.17	1.15	1.18	< 0.001
15 to 19	4	0.0080	1.17	1.13	1.21	< 0.001
20 to 24	14	0.0300	0.99	0.97	1.01	0.3653
25 to 29	36	0.0628	1.03	1.01	1.06	0.006
30 to 34	46	0.0743	1.06	1.04	1.08	< 0.001
35 to 39	48	0.0906	1.09	1.07	1.12	< 0.001
40 to 44	20	0.0513	1.10	1.07	1.13	< 0.001
45 to 49	16	0.0385	1.09	1.05	1.12	< 0.001
50 to 54	20	0.0656	1.09	1.05	1.12	< 0.001
55 to 59	7	0.0313	1.06	1.02	1.11	0.010
60 to 64	5	0.0250	1.07	1.03	1.11	0.003
65 to 69	1	0.0058	1.08	1.04	1.12	0.002
70 to 74	1	0.0076	1.08	1.03	1.12	0.003
75 to 79	0	0	1.08	1.04	1.12	0.002
80 plus	0	0	1.08	1.04	1.12	0.002

**Table 6:** The annual average annual percentage change (AAPC) of HIV and AIDS reporting incidence (RI) at province level from 2004 to 2014

Province	AAPC in HIV RI NO. (95% CI)	AAPC in AIDS RI NO. (95% CI)
all	46.63(39.60,54.01)	20.57(-0.28,45.77)
Beijing	147.72(110.96,190.89)	19.10(0.30,41.43)
Tianjin	32.77(26.79,39.04)	8.86(-0.03,18.55)
Hebei	11.21(7.41,15.15)	2.86(-0.20,6.02)
Shanxi	16.01(10.88,21.37)	3.53(-1.09,8.36)
Inner Mongolia	21.87(15.70,28.37)	2.67(-0.30,5.72)
Liaoning	48.83(34.92,64.18)	8.16(-0.07,17.07)
Jilin	22.82(12.82,33.71)	7.98(0.52,15.98)
Heilongjiang	27.90(18.92,37.55)	5.80(0.17,11.74)
Shanghai	31.74(15.58,50.17)	18.80(1.11,39.59)
Jiangsu	31.83(23.62,40.59)	9.46(0.11,19.68)
Zhejiang	48.81(41.75,56.23)	15.29(0.90,31.72)
Anhui	14.08(6.97,21.66)	7.94(-1.55,18.33)
Fujian	34.19(21.69,47.97)	7.78(0.61,15.45)
Jiangxi	25.97(17.85,34.65)	10.12(0.20,21.03)
Shandong	11.35(7.77,15.05)	2.01(-0.07,4.14)
Henan	9.33(0.54,18.89)	3.81(-10.34,20.20)
Hubei	21.84(14.15,30.04)	8.98(-0.02,18.79)
Hunan	35.62(26.70,45.16)	21.15(0.08,46.66)
Guangdong	22.10(2.44,45.54)	18.22(-0.63,40.64)
Guangxi	111.12(-3.85,363.55)	180.62(-3.28,714.22)
Hainan	27.20(17.94,37.19)	10.71(-0.29,22.92)
Chongqing	241.88(170.14,332.67)	51.08(1.99,123.80)
Sichuan	283.60(215.28,366.71)	61.17(2.05,154.52)
Guizhou	114.41(67.61,174.28)	33.31(-0.38,78.40)
Yunnan	88.25(8.93,225.32)	123.90(-1.14,407.08)
Tibet	27.84(20.80,35.30)	2.63(-1.38,6.80)
Shaanxi	37.19(24.99,50.57)	7.85(0.06,16.24)
Gansu	17.47(11.90,23.32)	3.93(-0.08,8.11)
Qinghai	46.85(32.59,62.65)	10.04(-0.14,21.27)
Ningxia	28.86(21.83,36.30)	5.49(-0.37,11.70)
Xinjiang	228.15(27.38,745.38)	79.93(-7.84,251.29)

**Table 7:** The average annual percentage change (AAPC) of HIV and AIDS reporting incidence in each age group from 2004 to 2014

Age group	AAPC in HIV RI NO. (95% CI)	AAPC in AIDS RI NO. (95% CI)
1 to 4	5.79 (0.00,11.93)	2.71 (0.51,4.96)
5 to 9	4.61 (1.91,7.38)	2.12 (0.49,3.78)
10 to 14	0.78 (0.45,1.12)	0.40 (−0.29,1.11)
15 to 19	24.45 (17.49,31.83)	6.76 (2.90,10.76)
20 to 24	60.06 (40.85,81.89)	28.14 (13.20,45.05)
25 to 29	75.35 (19.60,157.10)	61.30 (28.23,102.89)
30 to 34	80.53 (31.84,147.21)	82.41 (42.28,133.85)
35 to 39	56.80 (36.34,80.34)	62.72 (34.71,96.55)
40 to 44	50.36 (43.08,58.00)	49.90 (28.89,74.33)
45 to 49	50.08 (41.38,59.31)	45.32 (32.90,58.90)
50 to 54	44.83 (29.59,61.86)	40.75 (20.70,64.13)
55 to 59	47.61 (36.81,59.25)	42.10 (26.14,60.09)
60 to 64	61.19 (49.18,74.17)	51.79 (32.37,74.05)
65 to 69	70.77 (55.94,87.00)	56.32 (32.97,83.78)
70 to 74	63.51 (49.86,78.40)	47.27 (29.78,67.10)
75 to 79	49.70 (40.50,59.51)	31.66 (20.64,43.70)
80 plus	34.71 (24.19,46.12)	16.94 (11.66,22.46)

**Table 8:** The average annual percentage change (AAPC) of AIDS reporting mortality (RM) at province level from 2004 to 2014

Province	AAPC in AIDS RM NO. (95% CI)
All	9.87 (8.60,11.15)
Beijing	1.97 (1.52,2.41)
Tianjin	2.25 (1.78,2.72)
Hebei	1.15 (0.67,1.63)
Shanxi	1.54 (0.66,2.43)
Inner Mongolia	0.92 (0.51,1.33)
Liaoning	2.52 (1.78,3.26)
Jilin	3.90 (2.75,5.07)
Heilongjiang	2.03 (1.63,2.43)
Shanghai	1.39 (0.96,1.83)
Jiangsu	2.65 (2.03,3.28)
Zhejiang	3.19 (2.71,3.66)
Anhui	3.34 (2.45,4.24)
Fujian	2.53 (1.33,3.75)
Jiangxi	3.70 (3.24,4.17)
Shandong	0.63 (0.46,0.80)
Henan	9.64 (−2.30,23.05)
Hubei	3.79 (2.94,4.64)
Hunan	11.00 (9.52,12.51)
Guangdong	7.93 (6.76,9.12)
Guangxi	103.49 (71.17,141.90)
Hainan	7.27 (6.12,8.44)
Chongqing	18.05 (14.34,21.88)
Sichuan	22.59 (18.48,26.84)
Guizhou	14.34 (11.83,16.91)
Yunnan	49.13 (41.35,57.33)
Tibet	1.08 (0.30,1.87)
Shaanxi	2.29 (1.76,2.82)
Gansu	1.50 (0.99,2.02)
Qinghai	3.38 (1.65,5.13)
Ningxia	2.50 (2.01,2.99)
Xinjiang	35.99 (28.49,43.93)

**Table 9:** The average annual percentage change (AAPC) of AIDS reporting mortality (RM) by age groups from 2004 to 2014

Age group	AAPC in AIDS RM NO. (95% CI)
1 to 4	0.72 (−0.15,1.59)
5 to 9	0.89 (0.34,1.44)
10 to 14	0.57 (0.01,1.12)
15 to 19	0.84 (0.55,1.12)
20 to 24	0.46 (0.09,0.84)
25 to 29	0.32 (−0.07,0.72)
30 to 34	0.45 (0.21,0.70)
35 to 39	0.44 (0.03,0.85)
40 to 44	0.24 (0.01,0.47)
45 to 49	1.15 (0.83,1.48)
50 to 54	4.93 (3.94,5.93)
55 to 59	13.11 (10.57,15.71)
60 to 64	20.44 (16.06,24.99)
65 to 69	16.78 (13.47,20.18)
70 to 74	13.19 (11.31,15.10)
75 to 79	11.80 (9.51,14.13)
80 plus	11.47 (9.45,13.52)

## Abbreviations

AAPC: Average annual percentage change
AIDS: Acquired immune deficiency syndrome
CI: Confidence interval
CISDCP: The China Information System for Disease Control and Prevention
EMD: Empirical mode decomposition
HAART: Highly active anti-retroviral therapy
HIV: Human immunodeficiency virus
OR: Odds ratio
RI: Reporting incidence
SARS: Severe acute respiratory syndromes
